# Optimal surgical strategy for hepatocellular carcinoma with portal vein tumor thrombus: A propensity score analysis

**DOI:** 10.18632/oncotarget.8642

**Published:** 2016-04-07

**Authors:** Yong-Fa Zhang, Yong Le, Wei Wei, Ru-Hai Zou, Jia-Hong Wang, Han-Yue OuYang, Cheng-Zuo Xiao, Xiao-Ping Zhong, Ming Shi, Rong-Ping Guo

**Affiliations:** ^1^ Department of Hepatobiliary Oncology of Sun Yat-sen University Cancer Center, Guangzhou, P.R. China; ^2^ State Key Laboratory of Oncology in South China, Guangzhou, P.R. China; ^3^ Collaborative Innovation Center for Cancer Medicine, Guangzhou, P.R. China; ^4^ Department of Ultrasonography of Sun Yat-sen University Cancer Center, Guangzhou, P.R. China; ^5^ Department of General surgery, Shenzhen Shajing Affiliated Hospital of Guangzhou Medical University, Shenzhen, P.R. China

**Keywords:** hepatocellular carcinoma, portal vein tumor thrombus, hepatic resection, en bloc resection, peeling off resection

## Abstract

**Objectives:**

The optimal surgical resection method for hepatocellular carcinoma (HCC) patients with portal vein tumor thrombus (PVTT) that maximizes both safety and long-term outcome has not yet been determined. The aim of this study was to compare the clinical outcomes following peeling off versus en bloc resection for PVTT.

**Methods:**

From 2005 to 2012, 252 patients with HCC and type I/II PVTT who underwent hepatic resection were divided into two groups according to whether they received en bloc resection (*n* = 113) or peeling off resection (*n* = 139). The clinical outcomes were compared before and after propensity score matching.

**Results:**

The propensity model matched 113 patients with en bloc resection for further analyses. After matching, overall survival (OS) and disease-free survival (DFS) rates were significantly increased in the en bloc group compared with the peeling off group (*p* = 0.011 and *p* = 0.015). A multivariate analysis indicated that en bloc resection independently improved both OS and DFS (HR = 1.471, 95% CI: 1.071-2.018, *p* = 0.017 and HR = 1.415, 95% CI: 1.068-1.874, *P*=0.016). The adverse events were not significantly different between the two groups. However, the peeling off group showed a significantly increased recurrence rate of vascular invasion compared with the en bloc group (23.9% *vs*. 9.7%, *p* = 0.005). Similar results were also demonstrated prior to the matched analysis.

**Conclusions:**

An en bloc resection is safe and confers a survival advantage compared with a peeling off resection in HCC patients with PVTT; thus, en bloc resection should be recommended as a standard treatment for these patients when possible.

## INTRODUCTION

Hepatocellular carcinoma (HCC) is the sixth most common cancer and the third leading cause of cancer-related death worldwide [1]. HCC has a propensity to invade the portal vein and cause tumor thrombosis [2], which has been demonstrated to be one of the most adverse prognostic factors for HCC [3, 4]. Approximately 12.5-39.7% of HCC patients demonstrate gross portal vein tumor thrombus (PVTT) at the time of diagnosis [5]. Moreover, a median survival of 2.7-4.0 months has been reported if the tumor is left untreated [6, 7]. HCC with PVTT remains a contraindication for liver transplantation because of the high rate of tumor recurrence as well as the severe shortage of donor organs [8]. Thus, surgical resection may remain the only therapies offering a chance for long-term survival in these patients, and many clinicians have proposed that surgery should be recommended when it is feasible [9–15].

However, to date, the surgical strategy for HCC with PVTT remains controversial, and few studies have addressed this problem [10, 16–18]. One study demonstrated the superiority of en bloc resection compared with peeling off resection for PVTT [19], although other investigators have questioned the validity of this finding because no differences in HCC recurrence or overall survival (OS) rates were identified between the two groups following resection with a curative intent [17, 18]. Unfortunately, the statistical power of these previously reported studies was limited, and no case-matched or randomized clinical trials have compared the outcomes of peeling off and en bloc resection for HCC with PVTT. Thus, the optimal surgical resection method for PVTT has not yet been determined.

In the present study, the outcomes of peeling off and en bloc resections with a curative intent for PVTT were investigated, using propensity score matching to select subjects in each group to minimize the bias that arises from patient backgrounds.

## RESULTS

### Patients

**Figure 1:**
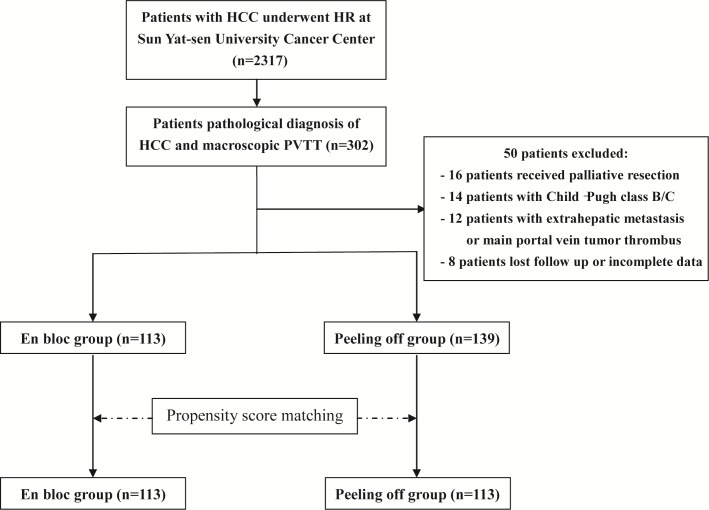
Flowchart of the study treatments

A total of 2,317 patients with HCC underwent hepatic resection at Sun Yat-sen University Cancer Center between 2005 and 2012. Three hundred and two patients had evidence of macroscopic vascular invasion and underwent primary resection. 50 patients did not fulfill the inclusion criteria and were excluded from the study. In the end, 252 patients (113 patients in the en bloc group and 139 patients in the peeling off group) were enrolled in the study (Figure 1).

Before matching, the median follow-up period was 15.3 months (range, 0.2-108.0 months) for the en bloc group and 9.6 months (range, 0.3-107.9 months) for the peeling off group. Compared with patients in the en bloc group, patients in the peeling off group exhibited higher rate of male (96% *vs*. 88%, *P* = 0.034), higher percentage of liver cirrhosis (57% *vs*. 42%, *P* = 0.023), higher AST levels (63.8 *vs*. 54.3 U/L, *P* = 0.068), greater MELD score (5.4 *vs*. 4.6, *P* = 0.034). The baseline patient characteristics before and after propensity score matching were summarized in Table 1. After matching, there was no significant difference between the en bloc and peeling off groups as shown in Table 1. The operative data are summarized in Table 2 according to the surgical resection method for PVTT. No significant difference was identified between the en bloc and peeling off groups with the exception of the time of Pringle's maneuver; the peeling off group had a significantly longer time of Pringle's maneuver compared with the en bloc group (*P* = 0.003).

**Table 1:** Baseline characteristics of patients before and after propensity score matching analysis

Characteristic		Before Matching		After Matching	
	En bloc group (*n* = 113)	Peeling off group (*n* = 139)	*P* Value	Peeling off group (*n* = 113)	*P* Value
**Epidemiology**					
Age (y)	49.1 ± 11.2	47.2 ± 10.8	0.168	47.7 ± 11.3	0.325
Gender (Male/female)	99/14 (88/12)	133/6 (96/4)	0.034	107/6 (95/5)	0.101
**Etiology**					0.313
Virus (HBV/HCV)	96 (85)	127 (91)	0.165	102 (90)	0.313
Others	17 (15)	12 (9)		11 (10)	
**Liver function**					
Liver cirrhosis (yes/no)	47/66 (42/58)	79/60 (57/43)	0.023	55/58 (49/51)	0.349
PLT (10^9^/L)	209.1 ± 83.6	201.1 ± 72.4	0.415	205.4 ± 70.7	0.722
PT (sec)	12.4 ± 1.4	12.6 ± 1.3	0.255	12.6 ± 1.3	0.390
AST (U/L)	54.3 ± 30.8	63.8 ± 48.2	0.068	56.0 ± 32.0	0.682
ALB (g/L)	41.2 ± 3.9	40.9 ± 3.8	0.626	40.9 ± 3.5	0.476
TBIL (mmol/L)	19.1 ± 34.0	23.5 ± 44.8	0.393	22.0 ± 43.0	0.579
MELD score	4.6 ± 3.1	5.4 ± 2.8	0.034	5.0 ± 2.7	0.226
**Tumor burden**					
Tumor size (cm)	8.5 ± 4.1	8.5 ± 2.9	0.900	8.6 ± 2.9	0.852
Tumor number (1/>1)	96/17 (85/15)	121/18 (87/13)	0.768	97/16 (85/15)	1.000
Tumor extent (unilobar/ bilobar)	106/7 (94/6)	127/12 (91/9)	0.625	104/9 (92/8)	0.795
AFP (≤/>200) (ng/mL)	38/75 (34/66)	39/100 (28/72)	0.414	32/81 (28/72)	0.472
Degree of PVTT (Type I/II)	48/65 (42/58)	57/82 (41/59)	0.915	47/66 (42/58)	1.000

Variables are expressed as the mean ± SD or no. (%), unless otherwise indicated;

PLT, platelet count; PT, prothrombin time; AST, aspartate aminotransferase; ALB, albumin; TBIL, total bilirubin; MELD, model for end-stage liver disease; AFP, alpha-fetoprotein; PVTT, portal vein tumor thrombosis.

**Table 2:** Operative results of patients in the en bloc and peeling off groups

Characteristic	Before Matching			After Matching	
	En bloc group (*n* = 113)	Peeling off group (*n* = 139)	*P* Value	Peeling off group (*n* = 113)	*P* Value
Surgical margin (<1/≥1 cm)	49/64 (43/57)	79/60 (57/43)	0.045	63/50 (56/44)	0.083
Hepatectomy (non-anatomical/anatomical)	48/65 (42/58)	77/62 (68/32)	0.056	63/50 (56/44)	0.062
Surgical time (min)	202.8 ± 75.6	206.2 ± 76.0	0.723	198.8 ± 69.6	0.676
Time of Pringle's maneuver (min)	17.0 ± 14.0	22.3 ± 13.8	0.003	22.4 ± 13.2	0.003
Estimated blood loss (mL)	697.3 ± 696.2	758.6 ± 737.4	0.502	745.1 ± 732.4	0.616
Red blood cell transfusion (mL)	283.6 ± 764.3	294.5 ± 908.6	0.920	308.2 ± 991.3	0.835
Tumor capsule (yes/no)	51/62 (45/55)	64/75 (46/54)	0.986	53/60 (47/53)	0.894
Edmondson grades (I, II/III, IV)	50/63 (44/56)	64/75 (46/54)	0.875	50/63 (44/56)	1.000

### Overall and disease-free survival

**Figure 2:**
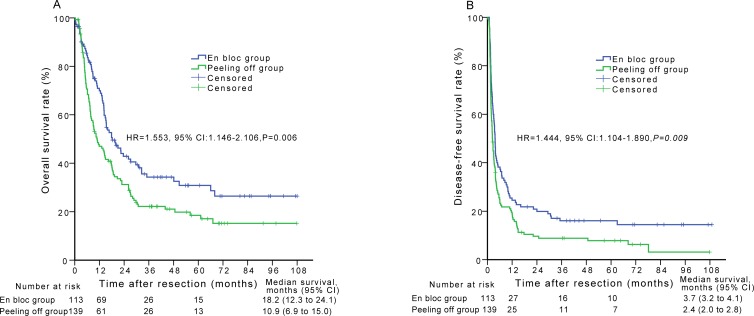
Overall survival (A) and disease-free survival (B) curves of patients in the en bloc group compared with those in the peeling off group before propensity score matching analysis

Before matching, 69/113 (61.1%) patients in the en bloc group and 106/139 (76.3%) patients in the peeling off group had died at the time of the primary survival analysis. The respective 1-, 3-, and 5-year OS rates were 68.9%, 34.3%, and 30.8% while the median survival time was 18.2 months for the en bloc group. In contrast, the respective 1-, 3-, and 5-year OS rates were 46.2%, 22.2%, and 17.1% while the median survival time was 10.9 months for the peeling off group. There were significant differences in the OS rates between the two groups (*P* = 0.006) (Figure 2A). The 1-, 2-, and 3-year disease-free survival (DFS) rates and the median DFS time were 23.6%, 19.9%, 16.1%, and 3.7 months, respectively, for the en bloc group and 18.8%, 8.9%, 7.9%, and 2.4 months, respectively, for the peeling off group. Among these patients, there were significant differences in DFS between the en bloc and peeling off groups (*P* = 0.009) (Figure 2B).

**Figure 3:**
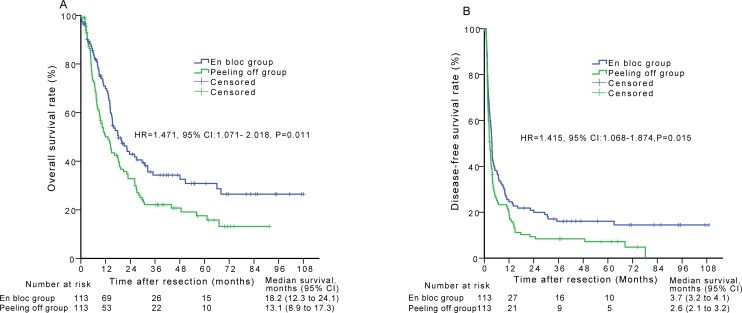
Overall survival (A) and disease-free survival (B) curves of patients in the en bloc group compared with those in the peeling off group after propensity score matching analysis

The 88/113 (77.9%) patients in the peeling off group had died at the time of the primary survival analysis after matching. The respective 1-, 3-, and 5-year OS rates and the median survival time were 49.1%, 22.1%, 15.8%, and 13.1 months, respectively, for the peeling off group. There were significant differences in the OS rates between the en bloc and peeling off groups (*P* = 0.011) (Figure 3A). The 1-, 2-, and 3-year DFS rates and the median DFS time were 19.6%, 8.4%, 7.2%, and 2.6 months, respectively, for the peeling off group. Among these patients, there were significant differences in DFS between the en bloc and peeling off groups (*P* = 0.015) (Figure 3B).

### Recurrence and treatment for recurrent HCC

Before matching, HCC recurrence was identified in 93/113 patients (82.3%) in the en bloc group and 127/139 patients (91.4%) in the peeling off group during the follow-up period. After matching, HCC recurrence was identified in 104/113 patients (92.0%) in the peeling off group. The recurrence rate in the peeling off group was higher than that in the en bloc group, although no significant difference was identified between the two groups (before matching, 91.4% *vs*. 82.3%, respectively, *P* = 0.099; after matching, 92.0% *vs*. 82.3%, respectively, *P* a significantly increased recurrence of vascular invasion compared with the en bloc group (before matching, 21.6% *vs*. 9.7%, respectively, *P* = 0.018; after matching, 23.9% *vs*. 9.7%, respectively, *P* = 0.005). The initial treatments used for recurrent HCC between the two groups are shown in Table 3 (before matching, *P* = 0.138; after matching, *P* = 0.211).

**Table 3:** Recurrence and treatment for the en bloc and peeling off groups

Variable	Before Matching			After Matching	
	En bloc group (*n* = 113)	Peeling off group (*n* = 139)	*P* Value	Peeling off group (*n* = 113)	*P* Value
Recurrence type*, n	93 (82.3)	127 (91.4)	0.099	104 (92.0)	0.081
Intrahepatic recurrence	77	109	0.089	89	0.098
Extrahepatic metastasis	29	42	0.510	34	0.553
Vascular invasion	11	30	0.018	27	0.005
**Treatment for the first recurrence, (n)**			0.138		0.211
TACE	49	78		58	
Resection	1	1		1	
Local ablation	3	2		2	
Systemic chemotherapy	3	0		0	
Sorafenib	1	3		3	

* Some patients had more than one type of recurrence.

### Mortality and morbidity

Mortality and complications are summarized in Table 4. Three patients in the en bloc group died as a result of abdominal infection and liver failure after the operation. Additionally, one patient in the peeling off group died from a postoperative biliary fistula infection accompanied by hepatic encephalopathy. However, both before and after matching, there were no significant differences between the two groups regarding postoperative complications and mortality.

**Table 4:** Mortality and complications for the en bloc and peeling off groups

Mortality and Complications	Before Matching			After Matching	
	En bloc group (*n* = 113)	Peeling off group (*n* = 139)	*P* Value	Peeling off group (*n* = 113)	*P* Value
Complications*, n					
I	7	10	0.753	9	0.604
II	18	17	0.398	15	0.572
III	10	12	0.952	11	0.819
IV	3	1	0.328	1	0.622
90-day mortality	3	1	0.328	1	0.622

* According to Dindo et al. Classification [38].

### Univariate and multivariate analyses of overall survival and disease-free survival for patients before and after the propensity score matching analysis

To investigate the influence of surgical resection variables on patient OS and DFS, the variables listed in Tables 1 and 2 were included in the univariate analysis. Before matching, in the multivariate analysis, the thrombectomy method (hazard ratio (HR) = 1.553; 95% confidence interval (CI), 1.146-2.106; *P* = 0.005) and degree of PVTT (HR = 1.418; 95% CI, 1.043-1.928; *P* = 0.026) were identified as independent predictors of OS (Table 5). The thrombectomy method (HR = 1.444; 95% CI, 1.104-1.890; *P* = 0.007) and the alpha-fetoprotein level (HR = 1.412; 95% CI, 1.057-1.885; *P* = 0.019) were identified as independent predictors of DFS (Table 5).

**Table 5:** Univariate and multivariate analyses of overall survival and disease-free survival for patients before propensity score matching analysis

Variable	Overall Survival				Disease-free Survival			
	Univariate analysis	Multivariate analysis			Univariate analysis	Multivariate analysis		
	*P*	*P*	HR	95% CI	*P*	*P*	HR	95% CI
Age (y), ≤/>50	0.087				0.024			
Gender (male/female)	0.826				0.107			
Etiology (virus/others)	0.077				0.155			
Liver cirrhosis (yes/no)	0.240				0.674			
PLT (10^9^/L), ≤/>100	0.622				0.339			
PT (sec), ≤/>13	0.209				0.860			
AST (U/L), ≤/>45	0.316				0.090			
ALB (g/L), ≤/>35	0.757				0.277			
TBIL (mmol/L), ≤/>17	0.650				0.888			
MELD score, ≤/>5	0.648				0.591			
Tumor size (cm), ≤/>5	0.024				0.212			
Tumor number, ≤/>1	0.207				0.491			
Tumor extent (unilobar/ bilobar)	0.559				0.762			
AFP (ng/mL), ≤/>200	0.078				0.024	0.019	1.412	1.057-1.885
Degree of PVTT (Type I/II)	0.035	0.026	1.418	1.043-1.928	0.124			
Surgical margin (<1/≥1 cm)	0.036				0.050			
Hepatectomy (non-anatomical /anatomical)	0.643				0.384			
Surgical time (min), ≤/>200	0.281				0.331			
Estimated blood loss (mL), ≤/>700	0.325				0.699			
Blood transfusion (mL), ≤/>300	0.099				0.102			
Time of Pringle's maneuver (min), ≤/>20	0.486				0.326			
Tumor capsule (yes/no)	0.521				0.475			
Edmondson grades (I, II/III, IV)	0.307				0.791			
Thrombectomy (en bloc/peeling off)	0.006	0.005	1.553	1.146-2.106	0.009	0.007	1.444	1.104-1.890

PLT, platelet count; PT, prothrombin time; AST, aspartate aminotransferase; ALB, albumin; TBIL, total bilirubin; MELD, model for end-stage liver disease; AFP, alpha-fetoprotein; PVTT, portal vein tumor thrombosis.

**Table 6:** Univariate and multivariate analyses of overall survival and disease-free survival for patients after propensity score matching analysis

Variable	Overall Survival				Disease-free Survival			
	Univariate analysis	Multivariate analysis			Univariate analysis	Multivariate analysis		
	*P*	*P*	HR	95% CI	*P*	*P*	HR	95% CI
Age (y), ≤/>50	0.128				0.036			
Gender (male/female)	0.926				0.127			
Etiology (virus/others)	0.175				0.368			
Liver cirrhosis (yes/no)	0.477				0.993			
PLT (10^2^/L), ≤/>100	0.350				0.560			
PT (sec), ≤/>13	0.318				0.710			
AST (U/L), ≤/>45	0.244				0.071			
ALB (g/L), ≤/>35	0.695				0.380			
TBIL (mmol/L), ≤/>17	0.849				0.559			
MELD score, ≤/>5	0.892				0.928			
Tumor size (cm), ≤/>5	0.016	0.025	1.435	1.046-1.969	0.185			
Tumor number, ≤/>1	0.242				0.591			
Tumor extent (unilobar/ bilobar)	0.246				0.519			
AFP (ng/mL), ≤/>200	0.204				0.024			
Degree of PVTT (Type I/II)	0.051				0.109			
Surgical margin (<1/≥1 cm)	0.067				0.049			
Hepatectomy (non-anatomical /anatomical)	0.819				0.280			
Surgical time (min), ≤/>200	0.577				0.488			
Estimated blood loss (mL), ≤/>700	0.187				0.571			
Blood transfusion (mL), ≤/>300	0.073				0.089			
Time of Pringle's maneuver (min), ≤/>20	0.391				0.368			
Tumor capsule (yes/no)	0.519				0.554			
Edmondson grades (I, II/III, IV)	0.208				0.720			
Thrombectomy (en bloc/peeling off)	0.011	0.017	1.471	1.071-2.018	0.015	0.016	1.415	1.068-1.874

PLT, platelet count; PT, prothrombin time; AST, aspartate aminotransferase; ALB, albumin; TBIL, total bilirubin; MELD, model for end-stage liver disease; AFP, alpha-fetoprotein; PVTT, portal vein tumor thrombosis.

The thrombectomy method (HR = 1.471; 95% CI, 1.071-2.018; *P* = 0.017) and tumor size (HR = 1.435; 95% CI, 1.046-1.969; *P* = 0.025) were identified as independent predictors of OS after matching as shown by the multivariate analysis. The thrombectomy method (HR = 1.415; 95% CI, 1.068-1.874; *P* = 0.016) was identified as an independent predictor of DFS.

## DISCUSSION

Prognosis remains poor for HCC patients with macrovascular PVTT [6]. Specifically, PVTT can lead to broad dissemination of the tumor throughout the liver and exacerbate portal hypertension, which may result in liver failure or life-threatening variceal bleeding [5]. Hepatic resection may be the last option for these patients. Several studies have reported that radical resection of the tumor and involved vessels can prolong survival and may even offer a chance of cure in selected cases [9, 20–22]. However, there is little consensus regarding the optimum treatment strategy for HCC patients with PVTT. In our study, we demonstrate that en bloc resection contributes to better OS and DFS after initial surgery in cases with HCC and PVTT. These findings suggest that en bloc resection may be superior to peeling off resection as an operative procedure for HCC patients with PVTT. Although three previous studies have compared the survival outcomes between en bloc and peeling off resections for HCC with PVTT, to our knowledge, our study comprises the largest study population and presents the longest follow-up data reported to date [17–19]. In addition, the current findings, which were obtained after balancing patient demographics, liver function reserves, and tumor characteristics between the en bloc and peeling off groups, provide important data that may be used to establish an optimal surgical strategy for the management of HCC patients with PVTT.

For HCC patients with PVTT, the en bloc technique, in which PVTT is resected together with the PVTT-bearing territory (internal wall of its portal vein), may be regarded as the only curative method available [18]. Potential reasons for misgivings regarding this technique are that the en bloc technique is a relatively complicated procedure and leads to a greater loss of liver parenchyma and blood. However, the present study demonstrated that en bloc and peeling off resections were not significantly different in terms of hospital mortality and morbidity, which is similar to previous studies [16, 17]. The peeling off technique, in which the PVTT is resected but the PVTT-bearing territory is preserved, may increase the risk of cancer cell residue on the portal venous wall. Indeed, because of a high incidence of intramural infiltration of cancer cells at the adhesion site of the portal vein cuff, the direct removal of thrombi in the portal vein could not be regarded as a curative resection for HCC [16]. Our study confirmed that the peeling off group showed a significantly increased recurrence of vascular invasion compared with the en bloc group (before matching, 21.6% *vs*. 9.7%, respectively, *P* = 0.018; after matching, 23.9% *vs*. 9.7%, respectively, *P* = 0.005). In addition, according to our multivariate analysis, en bloc thrombectomy was an independent prognostic factor for OS and DFS with the largest HR, which suggests that this procedure results in significantly better survival compared with peeling off thrombectomy. Thus, the entire thrombi-adhering segment of the portal vein should be resected in HCC patients with PVTT.

Anatomical resection is theoretically superior to non-anatomical resection because anatomical resection can eradicate the main tumor as well as micrometastases or microsatellite lesions along the portal tributaries; however, the clinical significance of these differences remains controversial [23, 24]. Several reports have demonstrated the effectiveness of anatomical resection for HCC in terms of postoperative recurrence and survival [23, 25–28]. In contrast, other reports have demonstrated no obvious superiority of anatomical resection compared with non-anatomical resection [24, 29, 30]. To date, the clinical benefit of anatomical resection even for early HCC remains controversial. For HCC patients with PVTT, anatomical resection showed no advantage compared with non-anatomical resection regarding postoperative outcomes in our study, consistent with previous studies [31, 32]. The resection margin has also been evaluated as a prognostic factor for long-term outcomes after HCC resection; however, the significance of this variable also remains controversial [19, 32, 33]. Our previous study explored patterns of intrahepatic micrometastasis using large pathological sections of liver resection specimens from 113 patients with solitary HCC [34]. We determined that the spread of the tumor satellite micronodules ranged from 0.1 to 0.8 cm, whereas the spread of the intravascular micrometastases ranged from 0.05 to 6.1 cm. We believe that dissemination *via* microscopic portal vein invasion cannot be controlled by extensive anatomic surgeries with wider resection margins [35]. In addition, patients with PVTT typically have injured livers; thus, extensive hepatic resection may cause serious postoperative liver failure, which is among the most common lethal complications of this procedure.

This study had several limitations, the most important of which was the lack of randomization. Instead, the treatment choices were determined in consideration of various clinical factors, which likely led to potential selection bias in our population. However, this bias was limited by the similar baseline characteristics between the two groups using propensity score matching analysis. Second, our study excluded patients who were diagnosed with PVTT that extended to the main portal vein (type III PVTT) or the contralateral portal vein. For these patients, resection remains controversial and is typically not recommended because of the high surgical mortality and low survival benefit [9, 15].

In conclusion, this study demonstrates that en bloc resection confers a survival advantage over peeling off resection for HCC patients with PVTT. Thus, en bloc resection represents the optimal surgical strategy for the management of HCC patients with PVTT and should be recommended as a standard treatment for these patients when possible.

## MATERIALS AND METHODS

### Study design

Between January 2005 and December 2012, treatment-naive patients with HCC who received first-line therapy with hepatic resection were retrospectively reviewed from a prospectively registered databank at Sun Yat-sen University Cancer Center. Only patients who had undergone hepatic resection with curative intent (R0) and had been pathologically diagnosed with macroscopic PVTT were screened for eligibility in the study (Figure 1).

The presence of PVTT was identified by preoperative imaging (Doppler ultrasound, contrast-enhanced computed tomography (CT), magnetic resonance imaging (MRI) or intra-operative ultrasound (IOUS)), naked eye observations during operation, and pathological examination. All patients diagnosed with HCC and PVTT were histopathologically confirmed in the resected specimens. According to previous reports [12, 36], the degree of PVTT was divided into 3 types based on the intraoperative findings. In type I, the tumor thrombi involve the segmental branches of the portal vein or above; in type II, the tumor thrombi extend to include the right/left portal vein; and, in type III, the main portal vein is involved.

The patients underwent the following examinations before surgery: routine blood chemistry tests, indocyanine green retention rate in 15 min (ICG-R15), color Doppler ultrasonography, and CT or MRI of the abdomen and chest. Patients were excluded from the study if they had one or more of the following conditions: (a) extrahepatic metastasis and main portal vein (type III PVTT) or contralateral portal vein tumor thrombosis; (b) Child-Pugh class B or C; (c) palliative tumor resection; or (d) incomplete data or loss to follow-up.

This study complied with the Health Insurance Portability and Accountability Act regulations and was approved by the Ethics Committee of the Cancer Center. Written informed consent was obtained from all patients in this study.

### Hepatic resection procedure

The techniques for hepatic resection were performed as our previously described [13, 36]. IOUS was routinely performed, and Pringle's maneuver was applied to occlude the liver's blood inflow. Anatomic hepatic resection with en bloc thrombectomy was our preferred surgical method for liver resection. As an alternative, non-anatomical resection was used in cases of intolerable en bloc wide resection. For each patient, the decision to perform major or minor resection was made pre-operatively on the basis of tumor location on preoperative imaging, liver functions of the patient, Child-Pugh status, indocyanine green retention at 15 min (ICG 15), and the likelihood of achieving an adequate resection margin. The treatment choice was ultimately determined by our multidisciplinary treatment team, which included radiologists, surgeons and oncologists [36]. The operative procedure and the location at which the portal branch should be ligated were determined preoperatively based on the liver functional reserve and the extent of the tumor itself, not the extent of the PVTT. The surgical management for PVTT was ultimately determined based on the findings of IOUS. If the portal branch could be ligated with a sufficient safety margin between its root and the tip of the PVTT, the en bloc technique was utilized. If the PVTT extended beyond the root of the portal branch to be ligated, the PVTT was extracted from the opened stump of the portal vein branch (peeling-off technique) [18]. With the en bloc method, macroscopic exposure of the PVTT did not occur. The portal vein was ligated at 2 different points with an adequate safety margin from the tip of the PVTT, and the section of the vein between the 2 ligations was divided (conventional en bloc technique). If a 2-point ligation was difficult because of a short distance to the branching site, a single ligation was placed at the branching site and the vein was carefully divided without injuring the PVTT during the final stage of liver transection (modified en bloc technique). With the peeling off technique, the portal venous wall was opened and separated from the PVTT and the PVTT was removed. The PVTT should be extracted before mobilization and transection of the liver to minimize the intraoperative migration of the tumor thrombus into the future remnant liver. After flushing with normal saline and confirming that no PVTT remained, the stump was closed with a continuous suture.

### Subsequent treatment

Recurrence after surgery was defined as the appearance of a new lesion with radiologic features typical of HCC, as confirmed by two or more imaging modalities. For patients who developed tumor recurrence, the treatment choice was determined by the characteristics of the recurrent tumor, the patient's request, and discussion among our multidisciplinary team [13, 37]. Conservative treatments were provided for patients with terminal HCC, Child-Pugh C liver function, or ECOG scores > 2.

### Follow-up

Follow-up examinations were conducted *via* laboratory findings (including serum alpha-fetoprotein (AFP), liver function, and blood tests), abdominal ultrasonography, and contrast-enhanced CT. The first CT was performed 4 weeks after surgery, every 3 months for the first year and every 6 months thereafter for a total of 60 months after treatment. The Complications was reported based on Clavien-Dindo classification [38]. Treatment mortality was defined as death within 90 days after surgery. The study was censored on August 31, 2015.

### Propensity score matching analysis

The demographic, preoperative and tumor variables were compared between the peeling off and en bloc groups. The operative approach was not randomly assigned; thus, there was a potential for confounding and selection bias between the two groups. Therefore, propensity score matching was conducted prior to comparisons of OS and DFS between the peeling off and the en bloc propensity score-matched groups.

Preoperative variables potentially affecting the outcomes were assigned propensity scores [39]. We employed a logistic regression model to estimate propensity scores, using the following baseline characteristics as covariates in the model: age (≤50/ > 50 y), gender (male/female), etiology (virus/other), liver cirrhosis (yes/no), platelet count (≤100/ > 100*10^9^/L), prothrombin time (≤13/ > 13 sec), aspartate aminotransferase (≤45/ > 45 U/L), albumin (≤35/ > 35 g/L), total bilirubin (≤17/ > 17 mmol/L), model for end-stage liver disease (MELD) score (≤5/ > 5), tumor size (≤5/ > 5 cm), tumor number (≤1/ > 1), tumor extent (unilobar/bilobar), AFP (≤200/ > 200 ng/ml), and degree of PVTT (Type I/II). Subsequently, a one-to-one match between the en bloc resection group and peeling off resection group was obtained by use of the nearest neighbor matching with the caliper width of 0.01 and without replacement [40]. Statistical analysis was performed using SPSS (IBM SPSS Statistics for Windows, Version 20.0. IBM Corp., Armonk, NY) and Propensity Score Matching for SPSS, version 1.0 (Felix Thoemmes, Cornell University/University of Tübingen).

### Statistical analysis

The primary outcome measures were OS and DFS. OS was calculated from the date of diagnosis until death or the end of the follow-up period. In this study, DFS was defined as the interval between the operation and the date of diagnosis of the first recurrence or the last follow-up. The secondary outcome measures included procedure-related complications and tumor recurrence type. The cutoff values of the continuous variables were based on those commonly used in previous studies or were dichotomized using normal reference values. For comparisons between the baseline variables, Student's t test for continuous variables and a χ2 test for categorical variables were used. Survival curves and univariate analysis were conducted using the Kaplan-Meier method, and the differences were analyzed *via* the log-rank test. The prognostic factors identified as significant in the univariate analysis (*P* < 0.1) were subjected to multivariate analysis with the Cox proportional hazards regression model. A statistically significant difference was set at *P* < 0.05.

## References

[1] Jemal A, Bray F, Center MM, Ferlay J, Ward E, Forman D (2011). Global cancer statistics. CA Cancer J Clin.

[2] Nakashima T, Okuda K, Kojiro M, Jimi A, Yamaguchi R, Sakamoto K, Ikari T (1983). Pathology of hepatocellular carcinoma in Japan. 232 Consecutive cases autopsied in ten years. Cancer.

[3] Poon RT, Fan ST, Ng IO, Lo CM, Liu CL, Wong J (2000). Different risk factors and prognosis for early and late intrahepatic recurrence after resection of hepatocellular carcinoma. Cancer.

[4] Shah SA, Cleary SP, Wei AC, Yang I, Taylor BR, Hemming AW, Langer B, Grant DR, Greig PD, Gallinger S (2007). Recurrence after liver resection for hepatocellular carcinoma: risk factors, treatment, and outcomes. Surgery.

[5] Minagawa M, Makuuchi M (2006). Treatment of hepatocellular carcinoma accompanied by portal vein tumor thrombus. World journal of gastroenterology.

[6] Llovet JM, Bustamante J, Castells A, Vilana R, Ayuso Mdel C, Sala M, Bru C, Rodes J, Bruix J (1999). Natural history of untreated nonsurgical hepatocellular carcinoma: rationale for the design and evaluation of therapeutic trials. Hepatology.

[7] Villa E, Moles A, Ferretti I, Buttafoco P, Grottola A, Del Buono M, De Santis M, Manenti F (2000). Natural history of inoperable hepatocellular carcinoma: estrogen receptors’ status in the tumor is the strongest prognostic factor for survival. Hepatology.

[8] Majno P, Mazzaferro V (2006). Living donor liver transplantation for hepatocellular carcinoma exceeding conventional criteria: questions, answers and demands for a common language. Liver transplantation.

[9] Minagawa M, Makuuchi M, Takayama T, Ohtomo K (2001). Selection criteria for hepatectomy in patients with hepatocellular carcinoma and portal vein tumor thrombus. Annals of surgery.

[10] Chen XP, Qiu FZ, Wu ZD, Zhang ZW, Huang ZY, Chen YF, Zhang BX, He SQ, Zhang WG (2006). Effects of location and extension of portal vein tumor thrombus on long-term outcomes of surgical treatment for hepatocellular carcinoma. Annals of surgical oncology.

[11] Aldrighetti L, Pulitano C, Catena M, Arru M, Guzzetti E, Halliday J, Ferla G (2009). Liver resection with portal vein thrombectomy for hepatocellular carcinoma with vascular invasion. Annals of surgical oncology.

[12] Shi J, Lai EC, Li N, Guo WX, Xue J, Lau WY, Wu MC, Cheng SQ (2010). Surgical treatment of hepatocellular carcinoma with portal vein tumor thrombus. Annals of surgical oncology.

[13] Peng ZW, Guo RP, Zhang YJ, Lin XJ, Chen MS, Lau WY (2012). Hepatic resection *versus* transcatheter arterial chemoembolization for the treatment of hepatocellular carcinoma with portal vein tumor thrombus. Cancer.

[14] Ikai I, Yamaoka Y, Yamamoto Y, Ozaki N, Sakai Y, Satoh S, Shinkura N, Yamamoto M (1998). Surgical intervention for patients with stage IV-A hepatocellular carcinoma without lymph node metastasis: proposal as a standard therapy. Annals of surgery.

[15] Poon RT, Fan ST, Ng IO, Wong J (2003). Prognosis after hepatic resection for stage IVA hepatocellular carcinoma: a need for reclassification. Annals of surgery.

[16] Wu CC, Hsieh SR, Chen JT, Ho WL, Lin MC, Yeh DC, Liu TJ, P'Eng FK (2000). An appraisal of liver and portal vein resection for hepatocellular carcinoma with tumor thrombi extending to portal bifurcation. Archives of surgery.

[17] Chok KS, Cheung TT, Chan SC, Poon RT, Fan ST, Lo CM (2014). Surgical outcomes in hepatocellular carcinoma patients with portal vein tumor thrombosis. World journal of surgery.

[18] Inoue Y, Hasegawa K, Ishizawa T, Aoki T, Sano K, Beck Y, Imamura H, Sugawara Y, Kokudo N, Makuuchi M (2009). Is there any difference in survival according to the portal tumor thrombectomy method in patients with hepatocellular carcinoma?. Surgery.

[19] Shaohua L, Qiaoxuan W, Peng S, Qing L, Zhongyuan Y, Ming S, Wei W, Rongping G (2015). Surgical Strategy for Hepatocellular Carcinoma Patients with Portal/Hepatic Vein Tumor Thrombosis. PloS one.

[20] Ohkubo T, Yamamoto J, Sugawara Y, Shimada K, Yamasaki S, Makuuchi M, Kosuge T (2000). Surgical results for hepatocellular carcinoma with macroscopic portal vein tumor thrombosis. Journal of the American College of Surgeons.

[21] Pawlik TM, Poon RT, Abdalla EK, Ikai I, Nagorney DM, Belghiti J, Kianmanesh R, Ng IO, Curley SA, Yamaoka Y, Lauwers GY, Vauthey JN (2005). Hepatectomy for hepatocellular carcinoma with major portal or hepatic vein invasion: results of a multicenter study. Surgery.

[22] Roayaie S, Jibara G, Taouli B, Schwartz M (2013). Resection of hepatocellular carcinoma with macroscopic vascular invasion. Annals of surgical oncology.

[23] Hasegawa K, Kokudo N, Imamura H, Matsuyama Y, Aoki T, Minagawa M, Sano K, Sugawara Y, Takayama T, Makuuchi M (2005). Prognostic impact of anatomic resection for hepatocellular carcinoma. Annals of surgery.

[24] Marubashi S, Gotoh K, Akita H, Takahashi H, Ito Y, Yano M, Ishikawa O, Sakon M (2015). Anatomical *versus* non-anatomical resection for hepatocellular carcinoma. The British journal of surgery.

[25] Sasaki K, Matsuda M, Ohkura Y, Hashimoto M, Watanabe G (2013). Anatomical *versus* nonanatomical resection in patients with hepatocellular carcinoma located in the left lateral segment. The American surgeon.

[26] Ueno S, Kubo F, Sakoda M, Hiwatashi K, Tateno T, Mataki Y, Maemura K, Shinchi H, Natsugoe S, Aikou T (2008). Efficacy of anatomic resection *vs* nonanatomic resection for small nodular hepatocellular carcinoma based on gross classification. Journal of hepato-biliary-pancreatic surgery.

[27] Eguchi S, Kanematsu T, Arii S, Okazaki M, Okita K, Omata M, Ikai I, Kudo M, Kojiro M, Makuuchi M, Monden M, Matsuyama Y, Nakanuma Y, Takayasu K, Liver Cancer Study Group of J (2008). Comparison of the outcomes between an anatomical subsegmentectomy and a non-anatomical minor hepatectomy for single hepatocellular carcinomas based on a Japanese nationwide survey. Surgery.

[28] Wakai T, Shirai Y, Sakata J, Kaneko K, Cruz PV, Akazawa K, Hatakeyama K (2007). Anatomic resection independently improves long-term survival in patients with T1-T2 hepatocellular carcinoma. Annals of surgical oncology.

[29] Marubashi S, Gotoh K, Akita H, Takahashi H, Sugimura K, Miyoshi N, Motoori M, Kishi K, Noura S, Fujiwara Y, Ohue M, Nakazawa T, Nakanishi K, Ito Y, Yano M, Ishikawa O (2015). Analysis of Recurrence Patterns After Anatomical or Non-anatomical Resection for Hepatocellular Carcinoma. Annals of surgical oncology.

[30] Okamura Y, Ito T, Sugiura T, Mori K, Uesaka K (2014). Anatomic *versus* nonanatomic hepatectomy for a solitary hepatocellular carcinoma : a case-controlled study with propensity score matching. Journal of gastrointestinal surgery.

[31] Pesi B, Ferrero A, Grazi GL, Cescon M, Russolillo N, Leo F, Boni L, Pinna AD, Capussotti L, Batignani G (2015). Liver resection with thrombectomy as a treatment of hepatocellular carcinoma with major vascular invasion: results from a retrospective multicentric study. American journal of surgery.

[32] Zhang T, Huang JW, Bai YN, Wu H, Zeng Y (2014). Recurrence and survivals following hepatic resection for hepatocellular carcinoma with major portal/hepatic vein tumor thrombus. Hepatology research.

[33] Ikai I, Hatano E, Hasegawa S, Fujii H, Taura K, Uyama N, Shimahara Y (2006). Prognostic index for patients with hepatocellular carcinoma combined with tumor thrombosis in the major portal vein. Journal of the American College of Surgeons.

[34] Shi M, Zhang CQ, Zhang YQ, Liang XM, Li JQ (2004). Micrometastases of solitary hepatocellular carcinoma and appropriate resection margin. World journal of surgery.

[35] Kang CM, Choi GH, Kim DH, Choi SB, Kim KS, Choi JS, Lee WJ (2010). Revisiting the role of nonanatomic resection of small (< or = 4 cm) and single hepatocellular carcinoma in patients with well-preserved liver function. The Journal of surgical research.

[36] Zhang YF, Guo RP, Zou RH, Shen JX, Wei W, Li SH, OuYang HY, Zhu HB, Xu L, Lao XM, Shi M (2015). Efficacy and safety of preoperative chemoembolization for resectable hepatocellular carcinoma with portal vein invasion: a prospective comparative study. European radiology.

[37] Luo J, Peng ZW, Guo RP, Zhang YQ, Li JQ, Chen MS, Shi M (2011). Hepatic resection *versus* transarterial lipiodol chemoembolization as the initial treatment for large, multiple, and resectable hepatocellular carcinomas: a prospective nonrandomized analysis. Radiology.

[38] Clavien PA, Barkun J, de Oliveira ML, Vauthey JN, Dindo D, Schulick RD, de Santibanes E, Pekolj J, Slankamenac K, Bassi C, Graf R, Vonlanthen R, Padbury R, Cameron JL, Makuuchi M (2009). The Clavien-Dindo classification of surgical complications: five-year experience. Annals of surgery.

[39] Brookhart MA, Schneeweiss S, Rothman KJ, Glynn RJ, Avorn J, Sturmer T (2006). Variable selection for propensity score models. American journal of epidemiology.

[40] Austin PC (2011). An Introduction to Propensity Score Methods for Reducing the Effects of Confounding in Observational Studies. Multivariate behavioral research.

